# Natural Gas-Derived Synthetic Fuels: A Comprehensive Review of Pathways for Carbon Offset and Sustainability

**DOI:** 10.1021/acsomega.5c05513

**Published:** 2026-01-28

**Authors:** Tala Katbeh, Tagwa Musa, Yasmin Abdelkarim, Hanif A. Choudhury, Mohamed S. Challiwala, Reza Tafreshi, Nimir O. Elbashir

**Affiliations:** † Chemical Engineering Program, 146313 Texas A&M University at Qatar, PO Box 23874, Doha, Qatar; ‡ Department of Multidisciplinary Engineering, Texas A&M University, College Station, Texas 77843, United States; § College of Science and Engineering, Hamad Bin Khalifa University, PO Box 34110, Doha, Qatar; ∥ Mechanical Engineering Program, Texas A&M University at Qatar, PO Box 23874, Doha, Qatar

## Abstract

The depletion of fossil fuels and the environmental impacts of conventional energy sources have accelerated the shift toward alternatives, with synthetic fuels—particularly those derived from natural gas—emerging as key solutions. gas-to-liquids (GTL) technology converts natural gas into ultraclean fuels and value-added chemicals, offering lower-emission alternatives to traditional crude oil-derived fuels. However, challenges, such as energy-intensive reforming stages, high capital costs, and the carbon footprint of natural gas feedstocks, hinder large-scale deployment. Additionally, the paraffinic composition and low aromatic content of GTL fuels present challenges in meeting engine performance and emissions targets without the use of tailored surrogate formulations. This review addresses these gaps by analyzing advancements in surrogate fuel development, with a focus on the limited kinetic data for high-molecular-weight iso-alkanes and cycloalkanes, which are critical for the accurate modeling of GTL-diesel and aviation fuels. Furthermore, the underexplored role of GTL-derived fuels in sustainable aviation fuel (SAF) strategies is evaluated, highlighting their potential as transitional low-carbon fuels. Novel insights include the integration of AI-driven computational methods and computer-aided molecular design (CAMD) to optimize fuel properties and process efficiencies. The review also synthesizes key technical, computational, and policy challenges and presents forward-looking research directions to guide the future development of GTL and SAF pathways. By bridging the gap between GTL chemistry, combustion modeling, and sustainability metrics, this Perspective outlines pathways for enhancing the role of GTL in the global energy transition.

## Graphical abstract

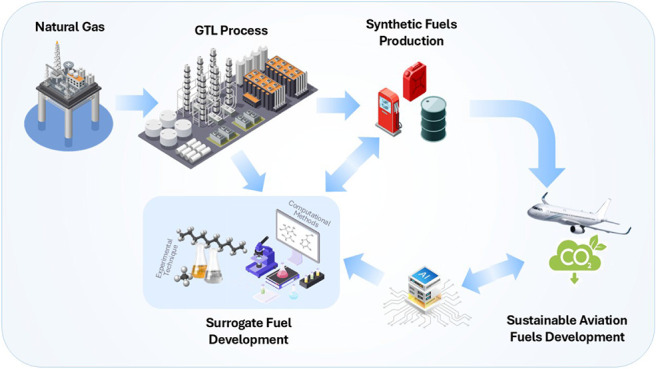

## 1 Introduction

The evolution of energy sources has significantly shaped historical advancements and societal transformation. Until the mid-19th century, biomass was the dominant global energy source, later replaced by coal during the Industrial Revolution. The discovery and utilization of crude oil revolutionized the energy sector, providing a more efficient and versatile fuel source for transportation and industrial applications.^1,2^ Today, global energy demand continues to rise, driven by population growth, economic development, and infrastructure expansion. Transportation alone accounts for 26% of total energy consumption, with 95% of this demand still being met by liquid fossil fuels. Projections indicate that liquid fuel consumption will increase by 7.6% between 2022 and 2050, underscoring the ongoing reliance on fossil fuels and the need for a transition to sustainable alternatives.^3^ As the world accelerates its efforts toward low-carbon energy solutions, synthetic fuels, hydrogen, and electricity are expected to play an increasingly significant role in the energy mix by 2035 and beyond.^4,5^ Achieving net-zero emissions requires a multifaceted strategy that incorporates low-carbon liquid fuels, refinery decarbonization, hydrogen-based alternatives, and electrification of transportation. However, a complete shift from liquid fuels remains impractical due to the extensive global reliance on internal combustion engines and existing fuel infrastructure. In this context, synthetic fuels, particularly gas-to-liquid (GTL) fuels, offer a viable transition pathway, delivering cleaner combustion, reduced emissions, and compatibility with current transportation and industrial systems.

GTL fuels, derived from natural gas conversion, have gained prominence as low-emission, high-quality liquid fuels, including diesel, kerosene, and naphtha. Compared with coal-to-liquid (CTL) fuels, GTL fuels exhibit lower sulfur content, reduced particulate emissions, and better air quality benefits. Moreover, their high energy density and seamless integration into the existing fuel infrastructure make them a practical alternative to crude oil-derived fuels. Additionally, advancements in surrogate fuel formulations improve combustion behavior and efficiency, paving the way for next-generation GTL applications in the automotive, aviation, and industrial sectors. Despite these advantages, economic, technological, and policy challenges remain. High capital costs, energy-intensive production processes, and carbon-intensive feedstocks have hindered the widespread adoption of GTL fuel on a commercial scale. However, recent advancements in computational modeling, machine learning, and AI-driven surrogate fuel formulation have the potential to enhance the performance and sustainability of GTL fuels. Additionally, emerging research into integrating GTL fuels with sustainable aviation fuel (SAF) pathways highlights new opportunities for decarbonizing the aviation sector.

While previous studies have examined GTL technology, they have primarily focused on technical fuel conversion processes or individual aspects of fuel properties.^6–14^ This review fills a critical gap by presenting a comprehensive and interdisciplinary assessment of GTL fuels within the context of the global energy transition. Specifically, this paper presents an integrated perspective on GTL fuels, encompassing production pathways, economic considerations, environmental impacts, and technological advancements in surrogate fuel formulation. It also examines the under-represented role of GTL fuels in SAF development, particularly their potential to reduce emissions in the aviation sector. Additionally, this review bridges the gap between fundamental GTL chemistry and real-world implementation challenges including scalability, infrastructure compatibility, and cost-effectiveness. The role of computational and AI-driven advancements in GTL fuel design, with a focus on machine-learning-based surrogate fuel optimization, is also examined. Furthermore, a comparative discussion of GTL fuels with other synthetic fuel pathways is presented, assessing sustainability, economic feasibility, and the lifecycle carbon footprint.

To justify the novelty and timeliness of this review, Table 1 summarizes representative review articles published in the past five years. These works have addressed various aspects of synthetic fuel development. Still, none provides a comprehensive integration of GTL technology with surrogate fuel formulation, AI-driven optimization, and its strategic role in SAF development.

**Table 1:** Representative Review Articles on Synthetic Fuels and GTL Technology in the Past Five Years

Year	Topic Reviewed	Key Focus	Covered Period	Reference	Key Limitations/Gaps
**2019**	Synthetic fuels for passenger vehicles	Environmental and techno-economic comparison of XTL pathways	Up to 2018	^15^	Limited discussion of GTL-specific combustion and surrogate formulation
**2020**	Fischer–Tropsch synthesis and GTL reactors	Reactor types and catalyst systems	Up to 2020	^16^	Focuses on FT synthesis, not downstream fuel applications or sustainability
**2021**	Synthetic fuels in transport decarbonization	Policy implications and fuel availability	Up to 2020	^17^	High-level policy view, minimal technical or surrogate fuel analysis
**2023**	Major synthetic fuels overview	Summary of FT, methanol, DME, SNG	Up to 2022	^18^	General overview; lacks depth in GTL process optimization or aviation fuel roles
**2023**	Synthetic fuels—origins and pathways	Overview of synthetic fuel types and sources	Up to 2023	^19^	Conceptual focus; does not address combustion behavior, surrogate modeling, or GTL-specific sustainability strategies
**2024**	GTL fuels in diesel engines	GTL properties, emissions, and blending	Up to 2023	^20^	Focused on diesel engine performance only; does not explore AI/CAMD or SAF pathways

Unlike previous reviews that treat GTL merely as a fuel conversion pathway, this paper evaluates its role in a decarbonized energy future and synthesizes key research, technology, and policy directions necessary for large-scale adoption. This review uniquely extends current understanding by (i) providing a structured comparison of GTL-BTL-CTL-PTL using sustainability and process-efficiency criteria reported in the literature, (ii) integrating AI and CAMD/CAMbD-based surrogate fuel workflows as reported in recent studies, including their typical validation steps and sources of model uncertainty, and (iii) framing GTL within the SAF transition via hybrid GTL-SAF blends and renewable-enabled e-GTL techno-economic pathways.

## 2 Synthetic Fuels Production

Synthetic fuels are derived from nonconventional feedstocks through chemical conversion processes, designed to emulate the chemical and physical properties of conventional petroleum-based fuels. Various X-to-Liquids (XTL) technologies exist, where “X” represents the feedstock source.^15,18,19,21–27^ The most common pathways include GTL, biomass-to-liquids (BTL), CTL, and Power-to-Liquids (PTL). A key advantage of XTL technologies is their ability to produce liquid fuels that are compatible with existing infrastructure while offering potential environmental benefits.^28^

BTL technologies convert biomass, including agricultural residues, forestry waste, and energy crops, into liquid fuels. The process involves gasification into synthesis gas (syngas), a mixture of carbon monoxide (CO) and hydrogen (H_2_), followed by catalytic conversion into hydrocarbons.^18^ Similarly, CTL technologies use coal gasification and Fischer–Tropsch (FT) synthesis to produce fuels such as diesel and kerosene.^16,29^ While these methods enable the utilization of diverse feedstocks, scalability and carbon emissions remain challenges, particularly for coal-based pathways.^30^

PTL, on the other hand, leverages renewable electricity to produce hydrogen via electrolysis, which is then combined with captured CO_2_ to synthesize liquid hydrocarbons. This pathway holds promise for carbon neutrality, especially when green hydrogen and direct air capture (DAC) technologies are utilized.^31^ However, PTL faces high production costs, substantial energy demands, and infrastructure development barriers, limiting its widespread large-scale adoption.^32–34^

Synthetic fuels offer several environmental benefits over conventional fossil fuels, including lower sulfur content and reduced particulate emissions.^17,35^ Additionally, they contribute to circular carbon economy goals by repurposing CO_2_ emissions into valuable energy carriers.^19^ However, the energy-intensive nature of their production means that their overall environmental impact depends on the source of the feedstock and the process efficiency.

Major synthetic fuels produced at an industrial scale, including FT fuels, methanol, dimethyl ether (DME), and synthetic natural gas (SNG), have been evaluated.^18^ FT fuels are synthesized by converting syngas into hydrocarbons. At the same time, methanol and DME can be derived from natural gas or biomass and used as fuels or chemical feedstocks. Additionally, SNG is produced by processing biomass or municipal solid waste into syngas, which is then upgraded to pipeline-quality gas. Their study highlighted the environmental benefits of synthetic fuels, particularly in terms of lower greenhouse gas emissions. Still, it emphasizes the need for more efficient and cost-effective production methods, particularly for fuels derived from biomass and waste.

As the global energy transition advances, there is a growing demand for alternative energy sources with a lower carbon footprint.^4,5^ This shift has spurred the development of fuel surrogates and the optimization of XTL fuel production techniques. Among XTL pathways, GTL technology has gained prominence due to its ability to produce ultraclean fuels with superior properties for modern engines.^36^ While BTL and CTL remain significant, their sustainability and carbon management strategies are under increasing scrutiny.^19^

### 2.1 Comparative Analysis of XTL Pathways

XTL pathways share common Fischer–Tropsch (FT) synthesis chemistry but differ significantly in feedstock quality, carbon intensity, scalability constraints, and energy requirements. To ensure comparability, carbon conversion efficiency (CCE), process energy efficiency (PEE) based on lower heating values (LHV) of fuels, and well-to-tank (WTT) greenhouse gas (GHG) emissions, excluding land-use change, were reported for each pathway from ranges compiled from published studies. The values reflect specific scenarios and should be interpreted as indicative comparisons rather than universal metrics. This is shown in Table 2 below.

**Table 2:** Indicative Performance of XTL Pathways

Pathway	Feedstock and Preprocessing	CCE (%)	PEE (%, Based on LHV)	WTT GHG Emissions (gCO_2_e/MJ fuel)	Key Bottlenecks
**GTL**	Natural gas → syngas → FT	52% ^37–39^	35–54.6% ^40,41^	101.0 ^42,43^	Limited by natural gas availability and methane slip
**CTL**	Coal → gasification (or direct liquefaction) → FT	28–34% ^37,39,44^	23.4–44.91% ^29,40,45^	194.8 ^42,43^	High CO_2_ burden, large capital cost, and high water use
**BTL**	Biomass → gasification → syngas → FT	43% ^37,39,46^	37.9–47.6%^47^	17.7 ^42,43^	Limited by biomass logistics and sustainability constraints
**PTL (e-GTL)**	Renewable electricity → H_2_ via electrolysis + CO_2_ capture → syngas → FT or other routes	68.4–88.6%,^48–50^ 98.6% ^51,52^	47–67.1% ^48,53–58^	11–28 ^42,43^	Highly dependent on low-cost renewable electricity

GTL benefits from clean methane feed and mature technology but exhibits a moderate CI due to natural gas extraction emissions and the energy-intensive reforming stage of the process. CTL offers large feedstock availability but is the most carbon-intensive pathway unless paired with high-capture CCS. BTL offers the lowest net GHG pathway but is constrained by the biomass supply, land use, and high capital cost of the process. As for PTL (or e-GTL), it provides the most significant long-term decarbonization potential but currently suffers from a low overall efficiency and high electricity costs.

The successful adoption of GTL, BTL, CTL, and PTL fuels depends on overcoming economic, technological, and policy-related challenges. Synthetic fuels have the potential to support global decarbonization goals, provided they are integrated with renewable energy sources and developed through advancements in conversion technologies.^13,19,59–65^ While previous studies have examined the various XTL pathways, this paper focuses on GTL technology, given its importance in the transition to low-carbon liquid fuels.

### 2.2 Overview of the GTL Process

The abundance of natural gas reserves in the Middle East, Iran, Uzbekistan, and Nigeria, along with the rise of shale gas extraction in North America, has paved the way for the development of GTL technology, a crucial pathway for producing ultraclean fuels via the FT process.^66^ GTL technology enables the conversion of natural gas into high-quality synthetic fuels, including diesel, kerosene, and naphtha. These fuels have lower sulfur content and reduced particulate emissions than conventional crude-derived fuels.

The GTL process consists of three primary stages^67–70^ (as depicted in Figure 1):

Reforming: Natural gas is converted into syngas. Depending on process conditions and economic considerations, this step can be achieved via steam methane reforming (SMR), partial oxidation (POX), or autothermal reforming (ATR).
FT Synthesis: The syngas undergoes FT polymerization, where metal catalysts (typically cobalt- or iron-based) facilitate the formation of long-chain hydrocarbons, producing a synthetic crude (syncrude).
Product Upgrading: The syncrude is refined into various fuels and valuable chemicals, including synthetic diesel, aviation fuels, lubricants, and waxes..

**Figure 1:**
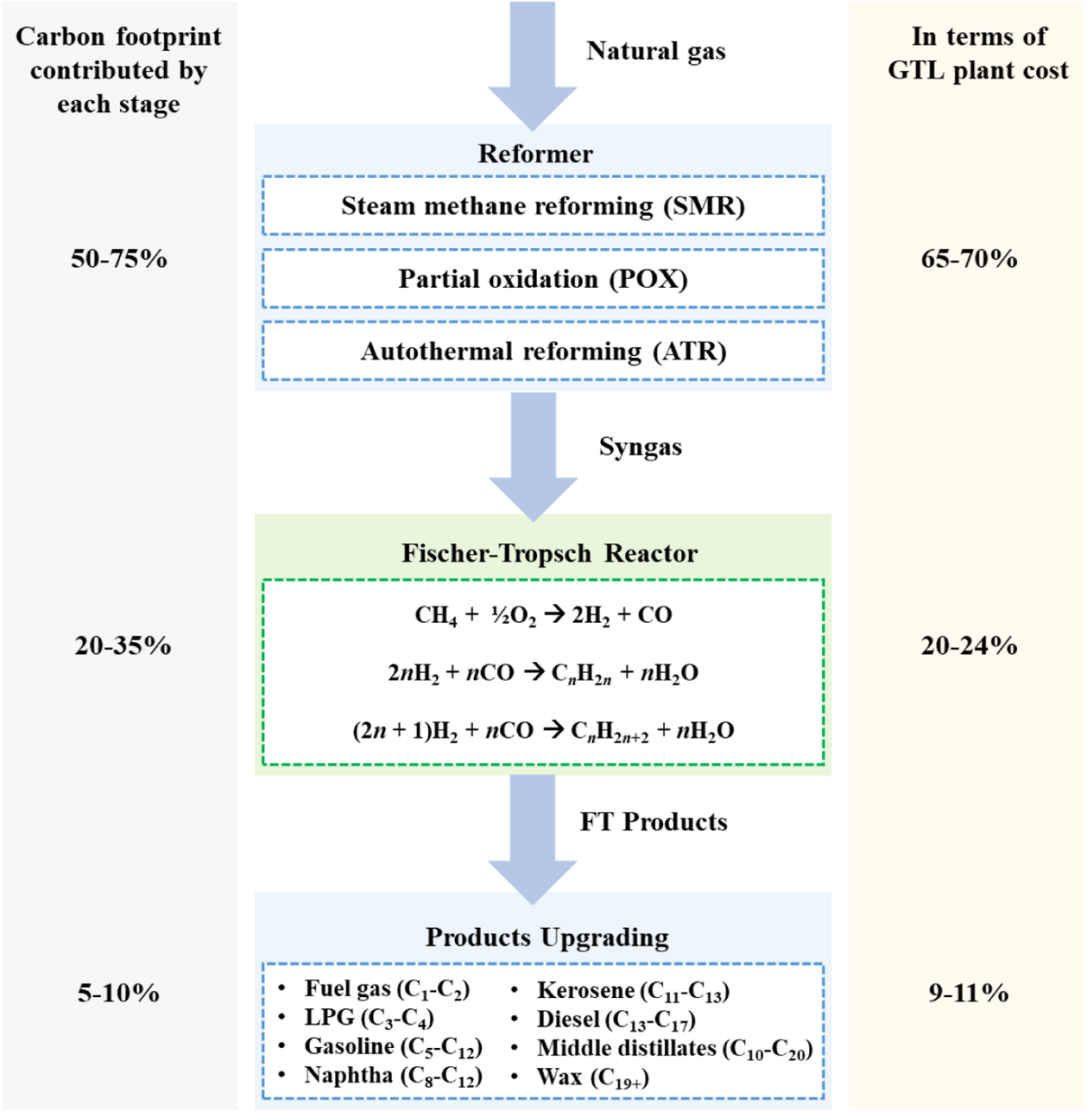
Stages of the GTL process.

Although GTL technology produces ultraclean fuels, it is highly energy-intensive, particularly during the reforming stage, which accounts for 50–75% of the process’s total carbon footprint and 65–70% of GTL plant capital costs.^71^ Optimizing process efficiency and integrating carbon capture solutions are critical to enhancing GTL’s viability as a sustainable fuel source.

The demand for low-emission transportation fuels has driven interest in GTL, as strict environmental mandates favor sulfur- and aromatic-free synthetic fuels over conventional fossil fuels. However, GTL fuels present challenges in physical properties, such as lower density and bulk modulus, which can lead to slightly reduced torque, power output, and brake thermal efficiency (BTE) in unmodified internal combustion engines.^20^ This issue necessitates the development of systematic fuel surrogates to design GTL fuels that meet the industry specifications for modern transportation applications.

### 2.3 GTL Fuel Performance and Applications

Despite minor variations in physical properties, GTL synthetic fuels provide a sustainable alternative to conventional petroleum-derived fuels, offering clean combustion with minimal modifications required for existing engines.^72^ Using GTL fuel blends is particularly promising, as synthetic fuel mixtures improve engine compatibility while reducing GHG emissions. However, widespread adoption of GTL fuels is contingent on overcoming economic, technological, and societal barriers through advances in fuel formulation and process optimization.

Synthetic GTL diesel exhibits unique properties compared to conventional diesel, including lower density, viscosity, sulfur content, and aromatic fractions, all of which contribute to cleaner combustion and reduced emissions.^73–75^ Although it has a lower volumetric heating value, GTL diesel generally demonstrates higher thermal efficiency, allowing for reduced particulate and NO_*x*_ emissions without requiring significant engine modifications.^73^

Several studies have investigated the performance of the GTL fuel under various conditions. For example, one study investigated the effects of altitude on emissions, combustion timing, and engine performance using GTL, biodiesel, and conventional diesel.^73^ The results demonstrated that GTL’s high cetane number reduces ignition delay, improving combustion efficiency and lowering particulate emissions. However, altitude-related NO_*x*_ emission challenges were observed due to oxygen availability variations, which affected the performance of the exhaust gas recirculation (EGR) system.

Further research examined 14 GTL diesel samples with different cold-flow properties, showing that increased isomerization significantly improves low-temperature operability while maintaining density, cetane number, and viscosity stability.^75^ The study highlighted the effectiveness of cloud point and low-temperature flow tests (LTFTs) in evaluating the performance of winter-grade GTL diesel.

Blended fuel studies offer additional insights into the benefits of GTL combustion. One study compared GTL, biodiesel, and conventional diesel blends, noting that a 20% GTL blend (G20) offers superior combustion characteristics due to its lower density, lower viscosity, and higher cetane number.^74^ The study reported that G20 blends enhance fuel atomization, reduce brake-specific fuel consumption (BSFC), and improve brake thermal efficiency (BTE) relative to petroleum diesel. However, higher biodiesel fractions in GTL-biodiesel blends increase viscosity, adversely impacting the combustion performance.

Beyond diesel applications, GTL-derived synthetic kerosene is emerging as a key component in aviation fuel blends, particularly Synthetic Paraffinic Kerosene (SPK), which offers lower sulfur oxide (SO_*x*_) emissions and reduced particulate matter compared to conventional jet fuel. Similarly, GTL naphtha has been identified as a valuable feedstock for petrochemical and plastic production due to its low sulfur content and superior chemical properties.^74,75^

The relevant properties of the GTL fuel with their respective American Society for Testing and Materials (ASTM) standards are listed in Table 3.^13,20,71,74,76–79^

**Table 3:** Physicochemical Properties of GTL Fuels^a^

Property	ASTM Method	GTL Gasoline	GTL Diesel/Gasoil	GTL Kerosene
**Carbon Number**	-	C_4_–C_12_	C_9_–C_16_	C_8_–C_16_
**Boiling Range (°C)**	D7096 and D7213	35–220 °C	180–380 °C	150–325 °C
**Density at 20 °C (g/cm** ^**3**^)	D4052	0.720–0.775	<0.876	0.780–0.820
**Kinematic Viscosity at 40 °C (mm** ^**2**^ **/s)**	D7042	0.3	1.9–4.1	1.5–2.5
**Flash Point (°C)**	D93	–40 to −48	52–96	38–65
**Reid Vapor Pressure (RVP, Kpa)**	D6378	45–103		
**Research Octane Number (RON)**	-	87–93		
**Cetane Number (CN)**	D613		40–55	

a Values represent literature-reported typical ranges.^13,20,71,74,76–79^ Uncertainty arises from plant configuration, FT cut range, and instrument repeatability (typically ± 2–5%).

### 2.4 The Formulation of GTL Surrogate Fuels to Understand the Combustion Behavior

Surrogate fuels are mixtures of well-characterized chemical compounds designed to replicate the combustion behavior of real fuels while simplifying experimental and computational investigations.^80^ These mixtures are crucial for studying fuel chemistry, ignition characteristics, and emissions, particularly in the design of next-generation fuels with enhanced efficiency and environmental performance.

In GTL fuel development, surrogates play a crucial role in optimizing fuel formulations to address challenges associated with paraffinic composition and combustion behavior. The hydrocarbons produced in the GTL process are predominantly paraffinic, meaning they lack the complex molecular structures found in conventional crude oil-derived fuels. This absence of aromatics and cycloalkanes impacts combustion characteristics such as density, ignition delay, and flame propagation.^69,81–86^

To bridge this gap, researchers employ fuel blending strategies to enhance combustion stability, while ensuring that GTL fuels meet industry performance standards. Numerous studies have demonstrated that fuel composition directly influences key combustion properties, such as autoignition, spray formation, evaporation, and emissions.^86–91^ Additionally, the selection of appropriate surrogate compounds is critical in balancing chemical and physical properties, ensuring that synthetic fuels align with real-world engine requirements.^92^

Surrogate fuel formulations also support computational modeling and experimental validation, enabling the more efficient optimization of GTL fuels for transportation, aviation, and industrial applications. Recent advancements have expanded systematic methodologies for screening and selecting surrogate components, integrating thermodynamic data, reaction kinetics, and empirical testing.^92,93^ Formulating accurate surrogate fuels is paramount to reducing computational complexity and minimizing experimental testing costs when screening multiple potential fuel formulations. Sophisticated computational models have been developed to identify the best surrogate candidates based on chemical kinetics, thermodynamic properties, and ignition behavior, enabling researchers to optimize fuel performance efficiently.

### 2.5 Advances in the Development of Diesel and Gasoline Fuel Surrogates

#### 2.5.1 Experimental Investigations

Experimental studies are crucial for validating surrogate fuel formulations and ensuring that they accurately represent GTL-derived fuels in terms of their physical properties, combustion efficiency, and emissions performance. One study developed and tested five surrogate fuel blends for GTL-derived diesel, examining their physicochemical properties and combustion performance.^94^ The study found that aromatic compounds enhanced fuel density; however, this improvement came at the cost of a lower heating value (HHV) and a lower calculated cetane index (CCI), both of which are critical for combustion efficiency and emissions control. A key finding was that higher carbon chain length surrogates could potentially address these trade-offs, although further studies are needed to optimize their combustion profiles and emission characteristics.

Another study investigated the performance of diesel surrogates under direct injection compression ignition (DICI) conditions, focusing on blends of *n*-heptane, toluene, and cyclohexane.^95^ Their study highlighted the significant impact of aromatic compounds (e.g., toluene) on soot emissions primarily due to their density-enhancing effects. In contrast, high-volatility surrogates promoted premixed combustion, resulting in lower soot formation. These findings underscore the importance of carefully balancing the aromatic content and volatility in surrogate fuel formulations to optimize combustion efficiency while minimizing emissions.

A study on gasoline surrogate formulation utilized a combined experimental and computational approach, reducing reliance on trial-and-error experimentation.^88^ The research demonstrated that fuel blends with higher *n*-butane concentrations (5% vs the typical 2%) improved Reid Vapor Pressure (RVP), although challenges arose in terms of blending stability. The study successfully predicted target properties by incorporating computational modeling, but additional fine-tuning was necessary to optimize the viscosity and RVP values.

Taken together, these studies highlight a consistent challenge across GTL-derived diesel and gasoline surrogate formulations: the trade-off between optimizing critical fuel properties (e.g., density and volatility) and minimizing pollutant emissions, particularly soot. Aromatics, while useful for enhancing density, tend to increase the level of particulate formation. In contrast, highly volatile components can improve combustion but may compromise safety or affect fuel storage stability. This recurring theme highlights the complexity of designing surrogate fuels that strike a balance among combustion performance, emissions control, and compatibility with GTL fuel characteristics.

Moreover, no universal surrogate formulation exists, as the optimal blend varies based on application and engine operating conditions. The reviewed studies emphasize the importance of moving beyond single-property optimization toward more holistic approaches that account for multiple combustion and emissions parameters.

These experimental findings highlight the crucial role of surrogate fuel formulation in bridging the gap between GTL-derived fuels and their conventional petroleum-based counterparts. However, achieving this requires further studies that address the complex interplay among combustion profiles, emission characteristics, and blending strategies to ensure higher efficiency and lower environmental impact.

#### 2.5.2 Computational Techniques in Surrogate Fuel Design

##### 2.5.2.1 Simulation and Challenges in Developing Kinetic Models for Fuels

The development of surrogate fuel mixtures relies heavily on computational simulations to replicate the physical, chemical, and transport properties of real fuels. These simulations integrate chemical kinetic models, thermodynamic data, and combustion interactions to ensure that surrogate fuels accurately mimic conventional fuel behavior under various engine conditions. Group additivity methods are commonly employed to estimate compound effects on fuel properties, improving thermodynamic predictions and enhancing the accuracy of combustion models.^96–99^

Advancements in engine simulations and combustion kinetic modeling have enabled more precise predictions of fuel performance, yet significant challenges remain. The primary difficulty lies in the complexity of real fuels, which contain hundreds to thousands of hydrocarbon species. Developing detailed kinetic models for such complex mixtures requires high-performance computing resources and extensive experimental validation data sets. This makes it computationally expensive and time-intensive to create models capable of accurately simulating real-world fuel behavior.

Given these challenges, surrogate fuels have emerged as effective tools for simplifying simulations and capture critical combustion properties. A well-designed surrogate should accurately reflect key characteristics such as ignition delay, flame propagation, injection behavior, vaporization, and emissions tendencies.^86,100,101^ Figure 2 illustrates a standardized workflow for combustion testing in surrogate fuel development, synthesized from methodologies reported in the literature.^102–104^

**Figure 2:**
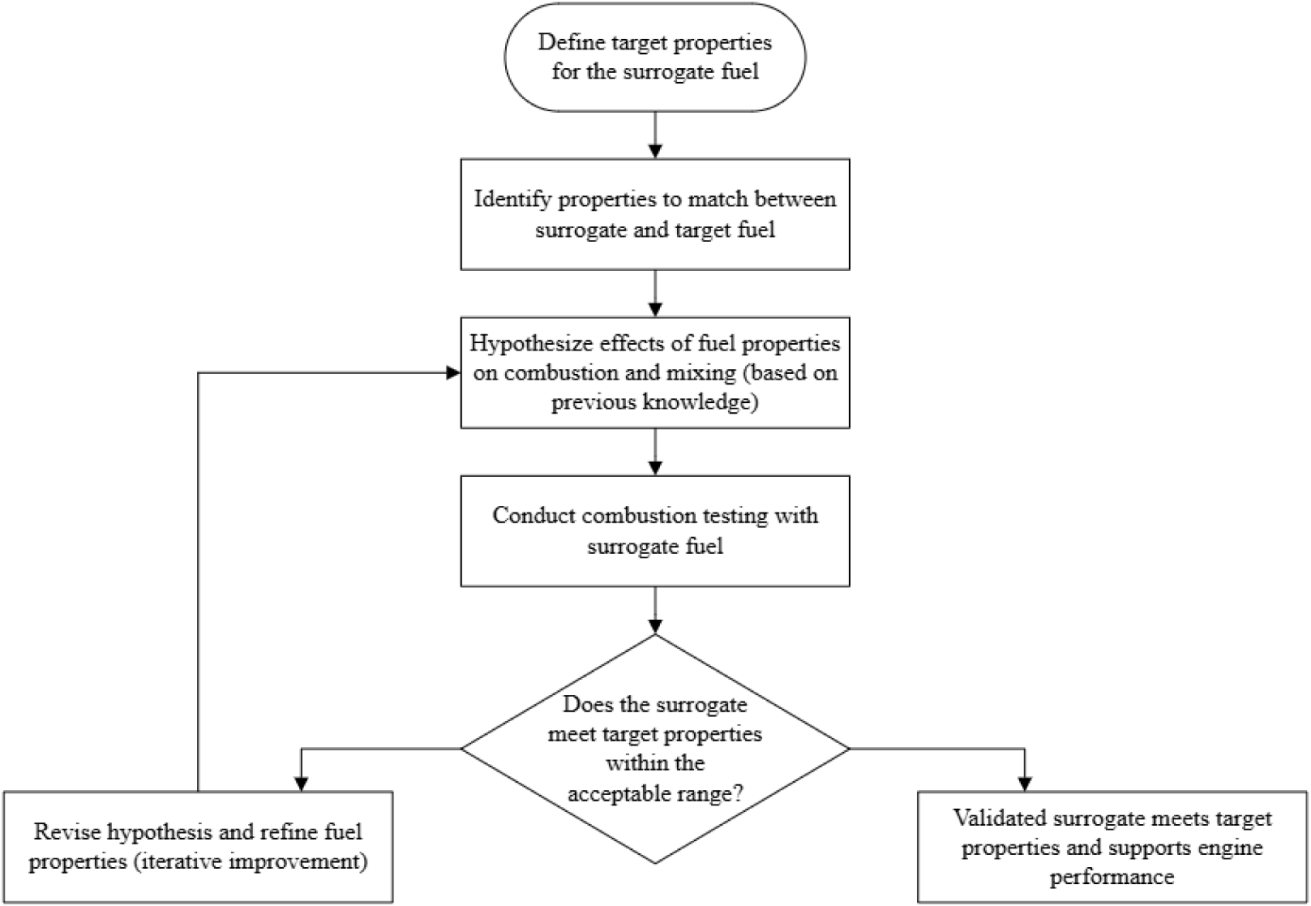
A standardized approach to conduct combustion testing.

Over the past two decades, kinetic models have become increasingly sophisticated, benefiting from improved computational resources and expanded reaction databases. Early work provided foundational insights into hydrocarbon oxidation mechanisms, covering *n*-alkanes, iso-alkanes, alkenes, cyclo-alkanes, and aromatics.^105^ More recent studies have expanded our understanding of iso-alkane kinetics, although gaps remain in the modeling of C_6_ to C_9_ compounds, which play a critical role in gasoline surrogate formulation.^106–112^

Previous works focused on C_8_ to C_16_ *n*-alkane kinetics for diesel surrogates, demonstrating that ignition delay times remain relatively consistent across this range.^113,114^ Further validation confirmed that all *n*-alkanes in this carbon range exhibit similar combustion behavior, reinforcing their suitability as diesel surrogate components.^115^

However, despite these advancements, several critical research gaps remain:

Limited kinetic data for C_10_ to C_12_ alkanes are highly relevant for jet fuel and gasoline surrogate formulations.^86,116^

Experimental studies primarily focus on shock tube experiments at an equivalence ratio of 1.0; however, higher equivalence ratios, which are more representative of real diesel engine conditions, require further investigation.
High-molecular-weight iso-alkanes remain underexplored, despite their importance in accurately formulating diesel surrogates.

Diesel fuels typically contain up to 30% cycloalkanes and 35% aromatics, which create challenges in terms of high-temperature volatility and combustion stability. Additionally, the high cost of pure cycloalkane samples has historically limited experimentation in this area. However, advancements in combustion modeling protocols and detailed kinetic models have facilitated more accurate predictions of ignition delay times and surrogate fuel combustion performance.

Several recent studies have significantly enhanced our understanding of cycloalkane and aromatic combustion kinetics, thereby contributing to the refinement of comprehensive surrogate fuel models.^101,115,117–142^ Despite these advancements, further research is necessary to develop more robust kinetic mechanisms that accurately capture combustion dynamics across a range of operating conditions.

The field has made significant progress in refining kinetic models, particularly for *n*-alkanes and cyclo-alkanes relevant to diesel surrogates. Yet, a persistent challenge remains in bridging fundamental kinetic insights with real-engine combustion behavior. While kinetic models for straight-chain alkanes are well-established, gaps in the kinetics of iso-alkanes and aromatic compounds create uncertainties in surrogate design, especially under high-pressure and low-temperature engine conditions. This requires integrated modeling strategies that combine detailed reaction mechanisms with advanced experimental validation campaigns to enhance the model reliability across practical combustion regimes.

##### 2.5.2.2 Advancements in Surrogate Fuel Design and Applications

The development of surrogate fuels has undergone significant evolution through kinetic modeling, experimental validation, and computational optimization. Several studies have refined modeling approaches, surrogate composition, and combustion characteristics, enabling more accurate predictions of the real fuel behavior.

Early work on surrogate fuels focused on establishing representative formulations for diesel and gasoline combustion studies. One study provided a comprehensive review of diesel surrogate fuel formulations, examining various approaches to mimic the properties of real fuels.^143^ A comparative study was conducted between gasoline and diesel surrogates, identifying key challenges in replicating the combustion behavior of conventional fuels.^87^ Advancements in kinetic modeling refined model reduction techniques to enhance the accuracy of surrogate fuel predictions in combustion simulations.^86^ More recent efforts optimized a diesel surrogate mixture to match boiling point and cloud point properties, integrating 6,407 species and 20,180 reactions in a kinetic model capable of replicating autoignition behavior under varying temperature and pressure conditions.^144^ Similarly, a systematic kinetic modeling framework was developed to demonstrate how reactive intermediate species influence ignition delay, flame propagation, and emissions.^145^

For gasoline surrogates, researchers have focused on optimizing the fuel component selection, antiknock properties, and chemical kinetics. One study classified various fuel components, including *n*-paraffins, iso-paraffins, olefins, naphthenes, and aromatics, to assess their impact on ignition delay and fuel knock resistance.^98^ A computational approach developed a numerical equilibrium combustion model, achieving an adiabatic flame temperature estimation accuracy of 0.1%, significantly improving fuel combustion performance predictions.^146^ Gasoline surrogate compositions and ignition delay have also been examined through Chemkin-Pro and Rapid Compression Machine (RCM) experiments, identifying gaps in kinetic data for oxygenated gasoline fuel combustion.^147,148^ Further enhancements in combustion modeling were introduced by developing a physical–chemical surrogate model that integrates continuous thermodynamic modeling (CTM) for droplet vaporization, thereby improving combustion simulations under high-pressure conditions.^149^

##### 2.5.2.3 Computational Tools in Surrogate Fuel Design

Significant advancements in machine learning (ML) and artificial intelligence (AI)- driven computational methods have benefited the optimization of surrogate fuel blends. These methods allow for more precise property predictions, fuel screening, and composition optimization. Fuel blend optimization integrates heuristics, databases, experiments, and computer-aided molecular design (CAMD).^93^ While traditional experimental screening is time-intensive, computational methods accelerate fuel formulation by predicting key properties, screening potential blends, and optimizing composition (as depicted in Figure 3).^150,151^ An AI-driven framework has been developed to optimize key fuel parameters, including research octane number (RON), motor octane number (MON), and yield sooting index (YSI). This effectively reduces computational costs while improving predictive accuracy.^152^ A related study introduced parametric reduced-order models based on Gaussian process kriging and deep neural networks, significantly reducing the computational burden of mixing and combustion simulations.^153^

**Figure 3:**
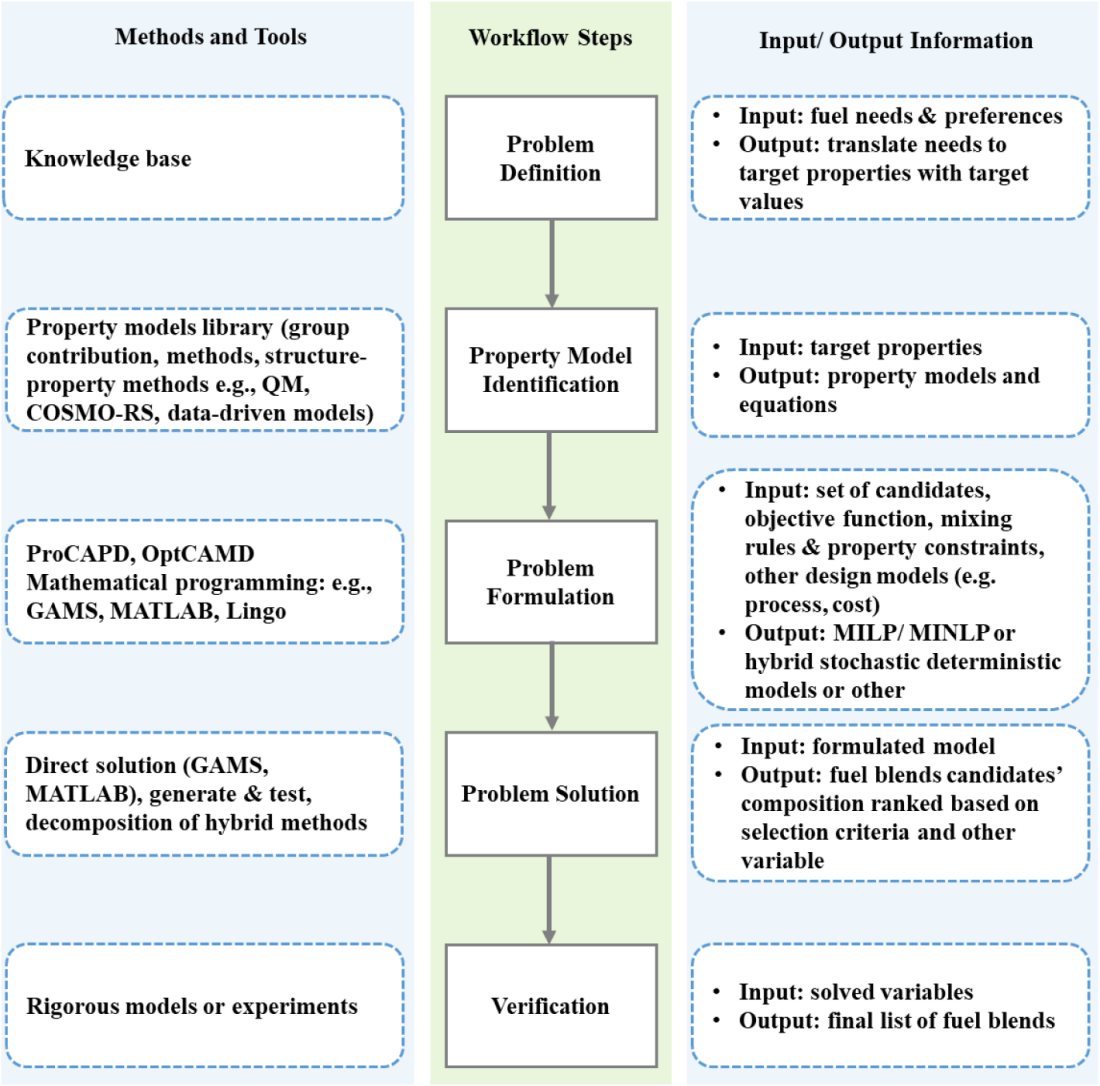
Conceptual workflow for computer-aided design of fuel blends, based on CAMD/CAMbD methodologies reported in the literature.^154^

##### 2.5.2.4 Computer-Aided Mixture and Blend Design (CAMbD)

Advances in CAMD and mixture blend optimization (CAMbD) have enabled the screening of chemical components to create optimized fuel formulations with desired properties.^155^ A Mixed Integer Nonlinear Programming (MINLP) approach was pioneered for optimizing fuel blends and later extended for GTL-derived diesel.^94,156^ This methodology was further refined by integrating phase-equilibrium-based property modeling to minimize the environmental impact of gasoline through the use of biobased additives.^154^ Additional advancements introduced a hybrid stochastic-deterministic optimization method that leverages genetic algorithms (GA) and gradient-based solvers to optimize fuel composition and gas absorption processes.^151^

However, solving CAMbD problems remains challenging, particularly due to the complexity of nonlinear equations. Several solution strategies have been reported, including direct solution methods,^157^ generate-and-test approaches,^158^ decomposition-based approaches,^159^ and hybrid models incorporating machine learning.^160^

ProCAPD, a comprehensive fuel design tool, integrates CAMbD with extended property databases for applications in lubricants, cetane index prediction, and CO_2_ emission analysis.^89^ A hybrid stochastic-deterministic optimization method has also been introduced, leveraging genetic algorithms (GA) and gradient-based solvers for fuel design and gas absorption process optimization.^151^

CAMbD and blend optimization studies have shifted the focus from property matching (e.g., density, viscosity) to multiobjective optimization frameworks. These methods not only target thermophysical properties but also increasingly account for life-cycle emissions and economic feasibility. However, the current generation of models still struggles with computational bottlenecks when simulating complex, multicomponent mixtures, which limits their application in industrial-scale surrogate fuel development.

##### 2.5.2.5 Quantum Mechanics and AI in Fuel Design

Quantum mechanical models (QM) have increasingly improved surrogate fuel selection and property predictions. Traditional group contribution (GC) methods offer fast, approximate estimations, but QM-based models like COSMO-RS provide higher accuracy by accounting for molecular-level interactions.^93,161^ COSMO-RS^162^ solvation models have also been successfully combined with CAMD to optimize solvent formulations and surrogate fuel compositions.^163^

AI-driven methods have further accelerated fuel formulation by automating the prediction of structure–property relationships. A deep learning-based quantitative structure–property relationship (QSPR) model was developed to improve the ability to identify optimal surrogate fuel candidates.^164^ Similarly, the ProCAPD database was expanded to integrate economic, environmental, and sustainability constraints into AI-driven fuel design models.^165^

These advancements demonstrate a growing trend toward hybrid modeling, where AI and quantum mechanics are combined to reduce uncertainty and computational time in surrogate modeling. Despite promising results, the lack of open-access, standardized data sets hampers the generalizability of these models, making cross-validation across different fuel types (e.g., GTL and biofuels) difficult. Moreover, most studies are constrained to lab-scale simulations, highlighting a critical need for model validation under real-world engine conditions.

##### 2.5.2.6 Challenges and Future Directions

Despite significant advancements in surrogate fuel modeling and optimization, several challenges remain:

Integrating molecular-scale simulations with full-scale engine models—Ensuring consistency between reaction kinetics models and real-world engine combustion behavior remains challenging.
Limitations in computational fluid dynamics (CFD) scalability—CFD is essential for product-process design, but nonlinear complexity and data inconsistencies restrict its scalability.^102^
Reliability of ML-assisted surrogate modeling—While machine learning has accelerated computation, the lack of standard benchmarking tools and data validation methods remains an issue.^160^
Environmental and regulatory considerations—Surrogate fuel research must align with sustainability metrics and regulatory compliance, requiring better chemical substitution frameworks.^166^
The need for expanded open-access surrogate fuel databases—Collaborative efforts across research fields will be crucial to enhancing the accuracy and accessibility of surrogate fuel data.^156^

Recent advancements in AI-driven surrogate fuel modeling have demonstrated promising results for GTL-diesel blend combustion simulations. A novel ML-genetic algorithm hybrid model, integrating CFD and AI techniques, has successfully analyzed flame speed, reaction kinetics, and emissions, highlighting the future potential of AI-driven fuel design methodologies.^167^ These emerging techniques are expected to bridge the gap between experimental validation and computational optimization, providing new insights into the development of next-generation fuels.

Future research must focus on holistic frameworks that integrate kinetic modeling, advanced AI algorithms, and full-scale engine simulations to realize the potential of computationally optimized surrogate fuels. Addressing this gap will enhance the predictive accuracy of fuel formulations and accelerate their deployment in practical combustion systems, aligning with low-carbon and regulatory targets.

High-fidelity CFD and CAMD-based fuel design simulations are computationally expensive due to the detailed chemistry and multicomponent mixture spaces involved. Recent literature proposes scalable surrogates based on reduced-order models (ROMs), physics-informed neural networks (PINNs), Gaussian process regression, and graph neural networks (GNNs) for mixture property prediction.^168–179^ These hybrid ML-ROM frameworks have demonstrated highly significant reductions in computational cost in combustion modeling, enabling real-time or near-real-time optimization.^168–170,180^ To the authors’ knowledge, these approaches have not yet been reported for GTL or e-GTL-specific surrogate fuels; however, the methodological frameworks are directly transferable, and future research can address this gap.

##### 2.5.2.7 Validation Strategy for AI/CAMD Surrogates

Recent advances in computational chemistry and ML have enabled the generation of candidate surrogate fuels through component selection, property prediction, and multiobjective optimization.^154,181–183^ However, reproducibility and generalizability remain dependent on robust validation against experimental benchmarks. Across the literature, validation generally occurs at three levels:

Thermophysical property validation: such as density at 15 °C, viscosity-temperature profiles, surface tension, vapor pressure, and ASTM D86 distillation curves.^88,145,184^

Combustion-relevant validation: including a certain derived number, ignition delay time measured in rapid compression machines (RCMs) and shock tubes, laminar flame speed, sooting tendency, and emissions markers.^185–189^

Application-oriented validation: in constant-volume combustion chambers, CFD simulations, and engine tests.^145,190–192^

While there are studies that use ML to predict fuel properties from molecular descriptors and use CAMD or systematic surrogate design with later validation against ignition delay, laminar flame speed, or engine experiments for generic jet/diesel surrogates, there is no broad, standardized benchmark set for GTL surrogate models yet. Some grounded suggestions in real practice include cross-validation against ASTM-type property targets using public data sets from conventional and FT fuel, in addition to validation against engine or combustor data, where available.

Regarding existing validation benchmarks, existing error margins, and existing gaps, Table 4 compiles selected surrogate fuel validation studies, highlighting the type of method (CAMD or AI-based), the experimental data sets used, and the reported error levels. It is essential to note that these studies do not target GTL or e-GTL-specific kerosene cut compositions yet; therefore, they are listed as methodological exemplars. In all cases, the core inputs are component identities and mixture compositions, while the outputs are fuel properties and combustion performance metrics. These methodologies demonstrate high transferability: once GTL-specific data sets (especially for iso-paraffins C_10_-C_12_) become available, these validation frameworks can be applied directly.

**Table 4:** Selected Validation Benchmarks for Surrogate Fuel and CAMD/AI-Designed Mixtures

Fuel Class and Context	Reference	Method Type	Validation Data Set	Reported Validation Performance
Gasoline-range surrogates for CFD in low-temperature combustion engines	^191^	ML-based property predictors and Bayesian optimization to select surrogate compositions from 15 species	Experimental volatility curves, Reid vapor pressure, and distillation data for several real gasolines	Mean absolute error for volatility/RVP/distillation properties is typically <3–5% relative to target fuels across tested cases; good agreement in multicomponent property space
Jet fuel surrogates focusing on sooting tendency	^193^	Bayesian multiple kernel learning (BMKL) model for predicting yield sooting index (YSI)	Measured YSI for base fuels and surrogate blends	BMKL achieves high correlation and low prediction error for YSI (R^2^ typically >0.9 over training data); used to guide surrogate selection with bounded sooting tendency
Generic liquid fuels (gasoline/diesel-like) with tailored properties	^152^	AI framework combining property predictors and optimization to design fuel mixtures with target combustion and thermophysical properties	Experimental property data sets for density, viscosity, octane/cetane surrogates, and other measured benchmarks	Reported errors for key properties (e.g., density and viscosity) are on the order of a few percent within the trained domain; framework shown to discover mixtures meeting multiple constraints simultaneously
Diesel surrogate fuels representative of FT and petroleum diesels	^184^	Systematic surrogate formulation based on detailed hydrocarbon analysis and kinetic mechanism development	Ignition delay measurements, cetane number, and other combustion metrics for target diesel fuels	Surrogate mixtures reproduce the derived cetane number and ignition behavior of target diesels to within experimental uncertainty across a range of conditions
Large diesel surrogate mechanism	^194^	Detailed kinetic mechanism and validation	Rapid compression machine (RCM) ignition delay and flow-reactor species data for surrogate vs target diesel	Surrogate reproduces total ignition delay and key species profiles over a broad range of equivalence ratios, temperature and pressure conditions; quantitative errors typically within experimental scatter
Multicomponent diesel surrogate fuels	^195^	Mechanism reduction and validation for multicomponent surrogates	Autoignition and oxidation data over multiple operating conditions	Reduced mechanisms maintain ignition performance within acceptable error while reducing computational cost

### 2.6 Advances in the Development of Aviation Fuels

The aviation sector is undergoing a major transformation in response to climate goals and emission reduction mandates. While aviation accounts for 2% of global CO_2_ emissions and 3.5% of total climate change impacts, international bodies such as the International Air Transport Association (IATA) and International Civil Aviation Organization (ICAO) have committed to net-zero aviation emissions by 2050.^196^ Achieving this goal requires scaling SAFs while exploring transitional low-carbon aviation fuels (LCAF) such as GTL-derived kerosene.

#### 2.6.1 Experimental Methods

Research on low-emission jet fuel alternatives has focused on fossil- and renewable aviation fuels. GTL-derived synthetic kerosene offers ultraclean combustion with low sulfur and particulate matter (PM) emissions, making it an attractive option for near-term emissions reductions. However, GTL is derived from natural gas through FT synthesis, so it does not achieve the CO_2_ reductions necessary to be classified as SAF under ASTM D7566 certification.^197^

Several experimental studies have sought to optimize synthetic aviation fuels, ensuring regulatory compliance and improved combustion performance. One study investigated the composition-property relationships of GTL-based synthetic jet fuels, evaluating density, kinematic viscosity, heat of combustion, and freezing point.^198^ Their study demonstrated that cyclo-paraffins are key in improving density, flash point, and cold-weather operability, making them essential for optimizing synthetic jet fuel formulations.

Further work focused on blending aromatics into GTL-derived jet fuel to improve lubricity and density, which are necessary for ensuring compatibility with existing aircraft engines.^199^ The optimization model revealed that toluene-based aromatic additions improved fuel performance, although excessive aromatic content may lead to higher soot emissions.

Additionally, an experimental campaign was conducted to enhance the lubricity of SPK derived from GTL fuels.^200^ The research identified linoleic acid as a promising lubricity additive, significantly improving the SPK performance without affecting its combustion properties.

Using modulated differential scanning calorimetry (MDSC), the specific heat capacity of conventional, synthetic, and biobased aviation fuels has also been compared.^201^ The findings indicated that biofuels exhibited the highest heat capacity, followed by FT synthetic fuels, underscoring the need for further refinement in thermodynamic property modeling to more accurately predict the performance of synthetic fuels.

More recent studies have also highlighted the importance of fuel composition in determining the compatibility of SAF with modern aviation systems. One critical factor is the dielectric constant, which influences aircraft fuel-gauging systems. One study examined the effect of fuel composition on dielectric properties, revealing that 100% paraffinic SAF lacks the necessary dielectric response for seamless integration into existing aviation fuel infrastructure.^202^ The findings suggest that blending aromatics or cycloalkanes into synthetic jet fuels, including those derived from GTL processes, may be necessary to maintain compatibility with ASTM-certified aviation fuels. Despite its ultraclean combustion properties, this aligns with previous research indicating that GTL-based kerosene may require specific additives to enhance density, lubricity, and sensor compatibility.

While these studies provide valuable insights into improving specific fuel properties, there is still no consensus on an optimal formulation strategy for GTL-derived SPK. In particular, the trade-offs between improving density via aromatic addition and the resulting increase in soot emissions remain a critical bottleneck in fully optimizing GTL-based aviation fuels. This highlights the need for multiobjective optimization approaches that balance fuel performance with environmental considerations.

Additionally, although additives like linoleic acid have shown promise in improving lubricity without degrading combustion quality,^203^ their long-term effects on engine wear and emissions in real-world aviation operations remain underexplored.

A recurring theme across these experimental efforts is the reliance on blending strategies to compensate for GTL-SPK’s inherent paraffinic composition. However, most studies focus on property-by-property optimization (e.g., density or lubricity) without holistically assessing how these blends affect full-scale engine performance or emissions trade-offs under high-altitude conditions.

#### 2.6.2 Computational Techniques

Computational models have played a crucial role in advancing aviation fuel research, particularly in the development of synthetic fuels and SAFs. Early studies provided an extensive review of kerosene fuel oxidation and combustion characteristics, forming the basis for kinetics modeling of aviation fuel surrogates.^204^ Further studies on aromatic oxidation pathways found that aromatic hydrocarbons, such as toluene, exhibit stronger hydrogen bonds, making them more resistant to oxidation than alkanes.^205^ This suggests that synthetic jet fuels must be carefully optimized to balance combustion efficiency and emission reduction by adjusting the aromatic content.

One study sheds the light on the role of fuel properties in enhancing the performance of jet engines.^206^ The research presents a mathematical model that accurately forecasts how aviation fuel properties impact end-user performance in terms of jet engine fuel consumption and emissions, and this computational tool can be used to evaluate the performance of SAF. In the study, fuel-property and engine-performance data were assembled from a range of published experiments and simulations, and a multiple linear regression (MLR) model was developed to correlate fundamental fuel properties (such as density, viscosity, and LHV) to changes in jet engine fuel use and emissions. Once trained and validated, the model was able to predict how alternative fuels would behave in a real engine environment using only their measured properties. This approach was then applied to several certified SAF pathways, showing that the property profiles of fuels such as HEFA and FT-derived blends translate into improved fuel-consumption trends and lower CO_2_ emissions compared with conventional Jet A-1. As such, the study demonstrates how relatively simple, data-driven models can serve as practical screening tools for comparing SAF options and understanding their operational impacts without requiring extensive engine-testing campaigns.

Recent computational studies have also focused on biofuel-kerosene blending and emissions modeling. One study conducted numerical simulations to investigate bioethanol-kerosene blends across blending ratios (0–50%) under varying pressure (0.3–1 MPa) and inlet air temperatures (350–650 K).^207^ Results showed that increasing the bioethanol ratio decreases soot emissions but leads to a marked exponential rise in NO_*x*_ when combustion chamber pressure and temperature are elevated. These findings elucidate the impact of biofuel on aviation fuel combustion and emissions certification.

In addition, large eddy simulation (LES), proper orthogonal decomposition (POD), and wavelet transform have been employed to model turbulent kerosene spray combustion.^208^ The analysis revealed that swirling flow structures have a significant impact on flame stability and combustion efficiency, underscoring the importance of optimizing the synthetic fuel-air mixing properties for aviation engines.

Optimization models have also been used to refine the synthetic fuel blending strategies. A linear programming model was developed to optimize the compositions of GTL-SAF blends.^199^ The results confirmed that carefully balancing paraffins and aromatics can enhance the density, flash point, and freeze point of synthetic jet fuel, ensuring regulatory compliance and compatibility with the existing aviation fuel infrastructure.

Experimental and computational approaches converge on balancing paraffinic and aromatic components within GTL-SPK blends. Yet, experimental studies focus on material compatibility and property compliance, while computational research drives the frontier in understanding combustion behavior and the formation of emissions. Bridging these two perspectives through integrated modeling frameworks and validation campaigns remains an unmet need in advancing GTL’s role as a viable transitional LCAF.

Integrating experimental insights with advanced computational tools is crucial for addressing the existing performance and emission challenges in GTL-SPK formulations. A more systematic exploration of GTL-SAF hybrid blending strategies, underpinned by empirical validation and high-fidelity simulations, could provide a comprehensive pathway for optimizing GTL-derived fuels in alignment with regulatory and environmental targets.

## 3 Outlook

### 3.1 GTL: A Pathway to Sustainable Energy Systems

Future research directions in GTL synthetic fuel combustion and surrogate fuel modeling should emphasize unexplored areas of study that contribute to a sustainable energy transition. The combustion behavior of surrogate fuels offers valuable insights into optimizing engine performance, controlling emissions, and designing effective exhaust after-treatment systems. These advancements are crucial in minimizing pollutant emissions and aligning GTL fuels with low-carbon energy policies.

Insights gained from surrogate fuel combustion behavior can inform the optimization of exhaust after-treatment systems and combustion control techniques, thereby minimizing pollutant emissions. A summary of the fuels’ requirements and details is listed in Table 5.

**Table 5:** A Comparison of GTL Gasoline, GTL Diesel, and GTL Kerosene Specifications^a^

Aspect	GTL Gasoline	GTL Diesel	GTL Kerosene
**Applications**	Automobiles, motorcycles	Heavy-duty vehicles, trucks, buses	Aviation, military, domestic heating
**Blending Requirements**	-Blending can result in fine-tuning of the fuel properties to meet specific requirements—In some cases, blending can be used to enhance combustion properties	- Aromatics are essential to boost fuel density GTL diesel is blended with conventional diesel in any ratio, depending on the fuel specifications	- The cyclo-paraffins and aromatics contribute to improving specific properties - Typically, synthetic fuels are blended with conventional jet fuels.
**Key Additives**	Antiknock agents		
**Energy Content (MJ/L)**	31.6	34.6	35.8
**Combustion Characteristics**	Rapid and controlled combustion, high ignition speed	Delayed and spontaneous combustion, high efficiency	Controlled, designed for high-altitude flight
**Regulatory Standards**	Euro 6 emission standards	Euro 6 emission standards	Aviation fuel specifications

a Energy content values typically vary within ± 5% depending on composition, isomerization, and blending strategy.^18,40,46,49^

A more comprehensive life-cycle assessment (LCA) of synthetic fuels is needed to evaluate their total environmental footprint, considering feedstock production, transportation, and emissions at each stage. Additionally, assessing economic feasibility is crucial, particularly in terms of large-scale production costs and government incentives to support market adoption. Industry-academia collaboration is critical in overcoming the technical, economic, and regulatory challenges that hinder the deployment of GTL fuels.

The sustainability of GTL pathways is strongly influenced by upstream natural gas leakage and the energy intensity of the reforming process. Multiple LCA reports indicate fossil GTL well-to-wheel GHG intensities between ∼90–150 gCO_2_e/MJ, comparable to or higher than conventional jet fuel (∼87–90 gCO_2_e/MJ).^209–212^ One study reports a value of 101 gCO_2_e/MJ for GTL Jet A.^42^ A key determinant is methane leakage: a rise from 0.2% to 2–3% can increase total GHG intensity by 12–30 gCO_2_e/MJ, consistent with NETL’s methane sensitivity analyses.^210^ Reforming accounts for 50–60% of GTL plant emissions, making autothermal reforming efficiency and CCS availability critical improvement levers.

Emerging research should also explore hybrid energy systems that integrate GTL synthetic fuels with renewable energy sources. While a single technology may not meet all transportation needs, combining synthetic fuels, green hydrogen, and electrification could significantly reduce GHG emissions.^213–220^ Integrating GTL fuels and renewable energy pathways will be crucial to advancing sustainable fuel production.

### 3.2 E-GTL Integration and SAF Implications

Integrating renewable energy into GTL production offers a transformative approach to sustainable fuel development, aligning with the vision of future refineries.^221^ Next-generation GTL processes could utilize CO_2_ captured from industrial emissions or DAC, along with green hydrogen produced through renewable-powered electrolysis to produce carbon-neutral liquid fuels.

By leveraging solar- and wind-powered electrolysis, GTL plants could reduce their carbon footprint while enhancing energy efficiency. Additionally, incorporating PTL technologies, which utilize CO_2_ and hydrogen to synthesize hydrocarbon fuels, can create low-emission synthetic fuels compatible with existing infrastructure.^31^

Future research should prioritize process optimization to minimize energy losses and carbon emissions within integrated GTL-renewable systems. Advancements in carbon capture, utilization, and storage (CCUS) and renewable hydrogen production will be crucial to achieving net-zero GTL fuel production by 2050.

The integration of renewable hydrogen and CO_2_ capture with FT synthesis enabled the e-GTL route. Mature PEM electrolyzers operate at a technology readiness level (TRL) of 8–9, whereas DAC systems are generally at a TRL of 6–7. FT synthesis integrated with renewable H_2_ and captured CO_2_ is typically assessed as TRL 4–6.^222–224^

Techno-economic assessments consistently indicate current e-kerosene production costs in the range of €4–6 per liter, dominated by electricity price, electrolyzer CAPEX, and CO_2_ capture. Concawe and LBST project future costs of €2–3 per liter by 2040–2050, contingent on reductions in renewable electricity price (<€30/MWh) and electrolyzer learning rates. Current PTL energy efficiencies are ∼−47% (LHV), potentially increasing to >50% with heat integration and high-efficiency electrolysis.^42,224,225^

On the other hand, unlike GTL, SAFs are derived from renewable and waste-based feedstocks, such as used cooking oil, municipal solid waste (MSW), and lignocellulosic biomass. SAF can reduce net CO_2_ emissions by up to 80% while maintaining the same performance and energy density as conventional jet fuels. Additionally, SAFs are certified as “drop-in” fuels, meaning that they can be blended with conventional jet fuel without requiring modifications to aircraft or fueling infrastructure.

Currently, seven SAF production pathways are certified under ASTM D7566, allowing for blending ratios of up to 50% with conventional jet fuel (Table 6).^197,226,227^

**Table 6:** Commercially Certified Sustainable Aviation Fuels Under ASTM D7566^a^ ^186,215,216^

Approved on	Technology	Blend	Feedstocks
2009	Fischer–Tropsch Synthetic Paraffinic Kerosene (FT-SPK)	50% Max.	Solid waste biomass, coal, and natural gas
2011	Hydro-processed esters and fatty acids synthetic paraffinic kerosene (HEFA-SPK)	50% Max.	Fatty acids, energy crops, oils, and greases
2014	Hydro-processed fermented sugars to synthetic isoparaffins (HFS-SIP)	10% Max.	Sugars
2015	Fischer–Tropsch synthetic paraffinic kerosene with aromatics (FT-SPK/A)	50% Max.	Solid waste biomass, coal, and natural gas
2016	Alcohol to jet synthetic paraffinic kerosene (ATJ-SPK)	50% Max.	Cellulosic biomass
2020	Catalytic hydro-thermolysis synthesized kerosene (CH-SK or CHJ)	50% Max.	Fatty acids, animal fats, oils, and greases
2020	Hydro-processed hydrocarbons, esters, and fatty acids synthetic paraffinic kerosene (HHC-SPK or HC-HEFA-SPK)	10% Max.	Bioderived hydrocarbons, fatty acid esters, and free fatty acids

a Feedstock yields and conversion efficiencies vary widely across commercial plants (typically ± 10–20% variability depending on feedstock quality and conversion process).

#### 3.2.1 Challenges and Future Research Directions for SAF Development

Various perspectives within the existing literature shed light on the potential of SAFs to reduce carbon emissions and foster a more sustainable aviation sector. Nevertheless, challenges persist, including production costs and optimizing infrastructure. The literature proposes different routes toward achieving zero aviation fuel, warranting careful evaluation to determine the most efficient strategy.

Despite strong industry momentum toward SAF adoption, several barriers remain:

High production costs: SAF production costs remain 2–5 times higher than conventional jet fuel, requiring policy support and subsidies.^228^
Feedstock limitations: Scaling the SAF supply requires diverse and sustainable feedstock sources, including waste-based and bioengineered solutions. Governments and policymakers are investing in expanding SAF production capacity, with targets set at 11.4 million tons annually by 2030 and 133 million tons by 2050. Hence, they need to endorse investments in SAF production and consumption.^229^
Infrastructure readiness: Although SAFs are drop-in compatible, scaling production and distribution networks remains a logistical challenge.^230^
  The availability of feedstocks alongside the processing infrastructure hinders SAF production capacity. However, despite these investments, SAF alone may not meet the demand for aviation fuel due to the limited availability of raw materials and land for cultivation.

Emerging research is addressing these challenges through:

Microalgae-based SAF: Studies suggest that microalgae-derived SAF reduces CO_2_ emissions while offering a sustainable, scalable feedstock option.^231–233^ However, current conversion processes require optimization of the costs.
Green hydrogen integration in SAF production: a study has highlighted that integrating renewable hydrogen in FT synthesis could increase SAF yields while lowering production costs by up to 18%.^228^
MSW and lignocellulosic biomass SAF: one study proposes that coprocessing plastic waste and food waste in catalytic hydroprocessing could enhance SAF yields while addressing waste management challenges.^234^
  Additionally, decentralized biorefinery models, where biomass is converted into syncrude locally before centralized refining, offer promising logistical and environmental benefits.^235^

#### 3.2.2 Positioning GTL in the Transition to SAFs

Although GTL-derived synthetic paraffinic kerosene (SPK) is certified as blends up to 50–50 with Jet A-1 in ASTM D7566 and offers superior combustion cleanliness, it does not qualify as an SAF because of its carbon footprint since GTL is fossil-derived and does not achieve the 80% CO_2_ reduction required for SAF classification. However, it still offers a lower-emission alternative to conventional petroleum-based jet fuels. From a policy perspective, ASTM D7566 and ICAO CORSIA sustainability frameworks restrict SAF eligibility to biomass-derived or captured-carbon pathways, excluding fossil-based GTL regardless of performance advantages. Therefore, SAF accreditation requires biogenic or captured CO_2_ carbon and a verified reduction in well-to-wheel GHG emissions. It is noteworthy to mention that GTL’s lack of aromatics also requires blending with fuels containing sufficient aromatics to satisfy elastomer swelling requirements. Nevertheless, GTL fuels can serve as a transition solution for low-carbon aviation fuels, supporting the development of SAF production capacity, as future blends could be an option for decarbonized GTL processes.^236^

To position GTL as a viable LCAF, future research should focus on:

Scaling electro-GTL (e-GTL) pathways using renewable hydrogen and CO_2_ capture to lower lifecycle emissions.
Blending GTL fuels with certified SAFs to provide immediate emissions reductions while leveraging existing infrastructure.
Advancing FT synthesis efficiencies for GTL-derived kerosene and biomass-based FT-SPK to create a more cost-competitive alternative.
Developing decentralized SAF production models that optimize feedstock logistics, cost efficiency, and environmental impact.

While SAFs will likely dominate long-term aviation fuel strategies, low-carbon synthetic fuels, such as GTL-derived kerosene, offer an intermediate solution in the transition toward net-zero aviation. Hybrid GTL-SAF blends offer a potential bridging option, simultaneously meeting compositional limits and reducing the well-to-wheel GHG intensity.

In parallel, recent studies have explored retrofitting existing GTL plants for low-carbon synthetic fuel production through the utilization of CO_2_ and the integration of renewable energy. One study evaluated three pathways: solar electrification, advanced reformer (e.g., CARGEN), and a hybrid configuration.^237^ Results showed that a hybrid configuration can achieve net-negative emissions (−138.9 g CO_2_e/bbl) while maintaining economic viability under moderate natural gas and carbon credit pricing.^236^ These findings highlight the potential of retrofitting strategies to bridge fossil-based infrastructure with net-zero SAF goals and underscore the importance of policy incentives and diversified revenue streams (e.g., multiwalled carbon nanotubes) for scalability and commercialization.

### 3.3 Societal Implications of GTL Fuels

The societal implications of GTL fuels present both opportunities and challenges. While their ultraclean combustion reduces tailpipe emissions, the production process is highly energy-intensive, contributing to significant CO_2_ emissions. FT synthesis and reforming steps account for a substantial portion of the lifecycle carbon footprint of GTL fuels, raising concerns about their overall environmental impact.

Despite these concerns, GTL fuels contribute to improved air quality by reducing SO_*x*_, NO_*x*_, and particulate matter (PM) emissions, which are linked to respiratory and cardiovascular diseases.^238^ This is particularly beneficial in urban centers. However, carbon-intensive production processes may negate these localized health benefits unless carbon capture and storage (CCS) technologies are integrated into GTL plants.^239^

Economically, GTL technology offers job creation and enhanced energy security in resource-rich regions such as the Middle East, Africa, and Asia by utilizing abundant natural gas reserves.^13^ However, high capital costs may restrict deployment to wealthier nations, exacerbating global energy inequities. Moreover, GTL production in regions with lax environmental regulations could lead to methane leakage from upstream operations, further contributing to climate change.^240^

To maximize sustainability, integrating renewable energy into GTL processes, such as utilizing green hydrogen in reforming stages, can help mitigate emissions.^241,242^ Policymakers and industry stakeholders must strike a balance between economic benefits and environmental responsibility, ensuring that GTL aligns with global sustainability goals.^243^

Table 7 provides a comprehensive overview of the key economic, policy, technological, and environmental challenges and opportunities for GTL fuel development.

**Table 7:** Key Challenges and Opportunities in GTL Fuel Development

Aspect	Challenges	Opportunities
**Economic Viability**	High production costs for GTL fuels, renewable energy, and green hydrogen production	Improving efficiencies in reforming and renewable energy infrastructure; cost reductions in electrolysis
**Policy and Regulations**	Lack of global incentives, inconsistent standards, and insufficient support for renewable integration	Alignment with international net-zero goals and climate agreements; government subsidies for renewables
**Scaling and Adoption**	Renewable energy storage limitations, infrastructure costs	Government subsidies and stakeholder collaborations
**Integration with Renewables**	High cost of green hydrogen and CO_2_ capture	Development of hybrid fuels combining GTL and renewables
**Technological Advancements**	Need for improving computational and experimental methods	Advancing AI/ML frameworks for GTL optimization

GTL fuels demonstrate excellent local air-quality performance, with independent engine/combustor tests reporting ∼70–90% reductions in PM and SO_*x*_ and lower NO_*x*_ in engine tests compared with conventional Jet A-1.^244–246^ However, global climate performance depends heavily on upstream natural gas leakage and reforming emissions. Without CCS or strict methane controls, fossil GTL can exhibit a higher well-to-wheel GHG intensity than conventional petroleum jet.^210^ These trade-offs underscore the need to strike a balance between local pollutant benefits and global climate criteria when evaluating GTL deployment.

## 4 Challenges and Future Perspectives

As global momentum builds toward net-zero energy systems, GTL fuels and synthetic fuel surrogates offer a compelling transitional pathway. However, realizing their full potential requires addressing a set of interrelated scientific, engineering, environmental, and policy challenges. This section synthesizes the critical bottlenecks identified across the literature and proposes actionable directions for future research and innovation.

### 4.1 Technical and Combustion Modeling Challenges

A key technical challenge lies in formulating surrogate fuels that strike a balance between performance and environmental outcomes. For both diesel and aviation applications, GTL-derived fuels are predominantly paraffinic, leading to excellent combustion cleanliness but suboptimal density, lubricity, and cold-flow properties. Adding aromatic and cyclo-paraffinic compounds improves these attributes but at the cost of increased soot formation. Current research still lacks consensus on optimal GTL-SPK blending strategies that holistically account for combustion stability, emissions, engine wear, and high-altitude performance.

At the modeling level, a notable gap exists in kinetic data for key species, particularly C_10_ to C_12_ alkanes and high-molecular-weight isoalkanes, which are crucial for accurate surrogate formulation. Experimental investigations are often limited to equivalence ratios of 1.0, while real engine conditions span wider operational envelopes. Moreover, no universal surrogate formulation has emerged due to varying application requirements across different engine types and operational regimes.

The most significant uncertainty in GTL combustion modeling arises from the limited kinetic data available for C_10_-C_12_ iso-paraffins, which comprise most of the composition of GTL kerosene. Existing detailed mechanisms extrapolate from smaller *n*-alkanes due to a lack of fundamental oxidation data. To address this gap, standard experimental tools include shock-tube ignition-delay measurements, rapid compression machine (RCM) ignition delay times, jet-stirred reactor species profiles, and laminar flame-speed measurements.^90,107,109^ Collaborative efforts—like those that established large *n*-alkane kinetics—would greatly improve mechanism accuracy for GTL-specific iso-paraffins.

Future work must expand the kinetic databases for underexplored compounds and emphasize model validation under practical conditions. Developing holistic, multiproperty optimization frameworks, rather than optimizing for individual fuel properties, will be critical to overcoming the current trade-offs in fuel design.

### 4.2 Computational Bottlenecks and Surrogate Design

Although computational tools such as CAMD and CAMbD have advanced rapidly, simulating multicomponent mixtures at scale remains computationally intensive. The complexity of surrogate formulation problems, often expressed through nonlinear equations, limits their applicability in industrial settings. Emerging AI/ML-based tools show promise, particularly in hybrid frameworks that combine genetic algorithms, surrogate models, and high-fidelity simulations. However, these approaches lack standardized benchmarking data sets and validation protocols.

To overcome these limitations, future research should prioritize:

The development of open-access surrogate fuel property databases, and
Standardized testing frameworks to assess ML-based predictive models, in addition to
Integrating the molecular-scale simulations with full-engine CFD models.

These efforts will accelerate the development of reliable surrogate fuels that align with regulatory and environmental performance metrics.

### 4.3 GTL Fuels in Sustainable Aviation: A Transitional Opportunity

GTL-derived kerosene, while not currently certified as an SAF,^197^ remains a valuable low-carbon alternative for the near-term decarbonization of aviation. Challenges remain in improving the density and lubricity of GTL-SPK without compromising the emission performance or sensor compatibility. Moreover, current formulations rely heavily on blending to compensate for deficiencies in paraffinic compositions, often optimized on a property-by-property basis.

Future efforts should focus on:

Multiobjective optimization frameworks that account for thermophysical, environmental, and economic trade-offs.
High-fidelity simulations of blended fuels under high-altitude and varied atmospheric conditions.
Experimental validation of additive effects (e.g., linoleic acid) on long-term engine performance.

Importantly, bridging computational modeling with real-world engine validation remains a critical unmet need in advancing the role of GTL in sustainable aviation fuels. Future research should integrate multiobjective optimization frameworks that simultaneously balance thermophysical properties, process efficiency, lifecycle emissions, and cost. Such frameworks can be expanded to optimize GTL-SAF blend ratios, FT synthesis conditions, and e-GTL system configurations within a unified decision space. This approach provides a systematic method for guiding next-generation GTL–SAF research, accelerating deployment, and ensuring that performance, environmental impact, and economic viability are co-optimized rather than addressed in isolation.^181–183,247–249^

### 4.4 Scaling SAF and Integrating GTL with Renewable Energy

Despite rapid progress, SAF faces persistent challenges in production costs, feedstock availability, and infrastructure readiness. GTL fuels, especially when combined with green hydrogen and carbon capture, can support the transition by acting as bridging solutions.

Future research should prioritize:

Development of e-GTL technologies that leverage renewable hydrogen and captured CO_2_.
Blending models that integrate GTL fuels with certified SAFs to maximize near-term emission reductions.
Process intensification strategies and decentralized biorefinery models to lower costs and improve logistics.

Such hybrid solutions offer a practical roadmap toward low-emission aviation fuel systems while SAF production matures.

In this context, methane plasmalysis has emerged as a promising approach to clean hydrogen production. Using plasma energy to split methane without emitting CO_2_, it can supply low-carbon hydrogen to GTL-FT systems when powered by renewables. The process also yields solid carbon with potential commercial value, improving synthetic fuel production’s overall economics and sustainability.^182^

### 4.5 Policy, Economics, and System-Level Integration

Ultimately, the large-scale deployment of GTL and synthetic fuels presents significant systemic challenges. These include high capital and operational costs, a lack of uniform policy incentives, and barriers to integration with renewable power infrastructure. Life cycle assessments of synthetic fuels are urgently needed to capture better emissions across feedstock production, transportation, and usage stages. Cross-sector collaboration, particularly between academia, industry, and policymakers, is crucial to unlocking GTL’s full sustainability potential.

Table 7 in this review outlines major challenges and corresponding opportunities in economic viability, policy, scaling, renewable integration, and technological advancement. Addressing these challenges requires scientific innovation, coordinated policy support, and investment in enabling infrastructure.

Policies such as carbon pricing, SAF blending mandates, renewable fuel standards, and tax credits significantly influence the competitiveness of GTL and e-GTL.^250,251^ Recent analyses indicate that high carbon prices (on the order of hundreds of dollars per tonne CO_2_ in the 2030s, and declining toward ∼$100/tCO_2_ by 2050) would be required to significantly narrow the cost gap between fossil jet and e-kerosene in the absence of other strong incentives.^225,252^ These instruments create long-term market signals for the PTL and advanced FT pathways. GTL equipped with CCS could benefit indirectly under regimes with sustained, relatively high carbon prices and stringent methane-leakage control, although the exact break-even carbon price remains scenario-dependent.

## 5 Conclusion

With population growth and economic development driving energy demand, particularly in Asia and Africa, transportation fuels will continue to play a central role, despite the rise of renewable electricity for mobility. With its global abundance and established infrastructure, natural gas serves as a critical transitional resource in the shift toward sustainable energy systems.

While natural gas-derived fuels offer cleaner combustion compared to conventional fossil fuels, their widespread adoption faces challenges in combustion performance, fuel properties, economic feasibility, and regulatory compliance. Addressing these limitations requires a holistic strategy that combines experimental research, advanced kinetic modeling, and AI-driven fuel formulation approaches to develop next-generation synthetic fuels.

Gas-to-liquid (GTL) technology stands out as a promising pathway for producing ultraclean synthetic fuels and value-added chemicals. When integrated with carbon capture and renewable energy sources, GTL can support the transition toward a circular carbon economy and help meet global net-zero emission targets. In the aviation sector, GTL-derived fuels offer drop-in compatibility and can serve as a transitional low-carbon alternative, while sustainable aviation fuels (SAFs) scale up.

This review highlights the significance of surrogate fuel formulation, life-cycle sustainability analysis, and process integration in shaping the future of GTL technologies. Advances in computer-aided molecular design, high-fidelity simulations, and hybrid SAF-GTL strategies will be crucial for optimizing performance and minimizing the environmental impact.

Emerging technologies, such as methane plasmalysis, could further decarbonize SAF production by supplying clean hydrogen without direct CO_2_ emissions. Their integration with GTL platforms offers a forward-looking pathway toward scalable, low-carbon aviation fuels.

Looking ahead, to guide the future design of synthetic fuels, particularly those derived from GTL processes, targeted research must focus on developing surrogate formulations that capture real-world combustion behavior under varying conditions for a specific set of physical and chemical properties that meet the requirements for fuels’ certification (e.g., ASTM D-1655 and ASTM D-7566 for aviation fuels). Key priorities include expanding kinetic data sets for high-molecular-weight iso-alkanes and cycloalkanes, refining AI-driven modeling tools for multiobjective optimization, and validating these models through high-pressure engine testing. Integrating fuel formulation with life-cycle emissions modeling and regulatory standards will be crucial for scale-up.

A systematic approach that combines chemistry, computation, and policy alignment will be vital to accelerating the development and deployment of cleaner, performance-optimized synthetic fuels for future energy systems.
